# Exploring Causal Links Between Gut Microbiota and Geriatric Syndromes: A Two-Sample Mendelian Randomization Analysis

**DOI:** 10.7150/ijms.94335

**Published:** 2024-07-22

**Authors:** Qiuru Yao, Ling Chen, Yuxin Cai, Changxi Li, Shuyang Wen, Chun Yang, Qi Zhang, Yuting Zeng, Shuqi Zheng, Jihua Zou, Guozhi Huang, Qing Zeng

**Affiliations:** 1 Department of Rehabilitation Medicine, Zhujiang Hospital, Southern Medical University, Guangzhou, China.; 2 School of Nursing, Southern Medical University, Guangzhou, China.; 3 School of Rehabilitation Sciences, Southern Medical University, Guangzhou, China.; 4 Department of Cardiology, Zhujiang Hospital, Southern Medical University, Guangzhou, China.; 5 Dongguan Key Laboratory of Stem Cell and Regenerative Tissue Engineering, Guangdong Medical University, Dongguan, Guangdong, China.; 6 Faculty of Health and Social Sciences, The Hong Kong Polytechnic University, Hong Kong, China.

**Keywords:** Geriatric syndrome, Gut microbiota, Mendelian randomization, SNPs

## Abstract

**Background:** Both observational studies and clinical trials have demonstrated a link between the gut microbiota and the geriatric syndrome. Nevertheless, the exact nature of this relationship, particularly concerning causality, remains elusive. Mendelian randomization (MR) is a method of inference based on genetic variation to assess the causal relationship between an exposure and an outcome. In this study, we conducted a two-sample Mendelian randomization (TSMR) study to fully reveal the potential genetic causal effects of gut microbiota on geriatric syndromes.

**Methods:** This study used data from genome wide association studies (GWAS) to investigate causal relationships between the gut microbiota and geriatric syndromes, including frailty, Parkinson's disease (PD), delirium, insomnia, and depression. The primary causal relationships were evaluated using the inverse-variance weighted method, MR Egger, simple mode, weighted mode and weighted median. To assess the robustness of the results, horizontal pleiotropy was examined through MR-Egger intercept and MR-presso methods. Heterogeneity was assessed using Cochran's Q test, and sensitivity was evaluated via the leave-one-out method.

**Results:** We identified 41 probable causal relationships between gut microbiota and five geriatric syndrome-associated illnesses using the inverse-variance weighted method. Frailty showed five positive and two negative causal relationships, while PD revealed three positive and four negative causal connections. Delirium showed three positive and two negative causal relationships. Similarly, insomnia demonstrated nine positive and two negative causal connections, while depression presented nine positive and two negative causal relationships.

**Conclusions:** Using the TSMR method and data from the public GWAS database and, we observed associations between specific microbiota groups and geriatric syndromes. These findings suggest a potential role of gut microbiota in the development of geriatric syndromes, providing valuable insights for further research into the causal relationship between gut microbiota and these syndromes.

## Background

Geriatric syndromes refer to typical age-related symptoms that gradually affect individuals' social and daily living abilities including difficulties in mobility, balance disorders, cognitive impairment, and incontinence, among others 1. Common geriatric syndromes include frailty, Parkinson's disease, delirium, insomnia, and depression. Frailty is a multi-system physiological state characterized by an individual's increased vulnerability, decreased physiological reserve capacity, and increased susceptibility to stressful events 2. The most widely used tools for assessing frailty in current clinical practice are the physical frailty phenotype proposed by Fried and the Frailty Index (FI) of accumulative deficits proposed by Rookwood *et al.* The Fried phenotype scale ranges from slowing of gait speed, loss of grip strength, weight loss (unexplained), perceived fatigue, and low physical activity to rate five dimensions of frailty 3, 4. Rookwood *et al.* 5, 6 developed the Frailty Index (FI) based on the theory of accumulative deficits, which is essentially a multidimensional approach to assessing frailty, covering physical functioning, multiple comorbidities, cognition, and psychosocial aspects. Parkinson's disease (PD) is the second most prevalent neurodegenerative illness worldwide, following Alzheimer's disease. There is a strong association between Parkinson's disease and geriatric syndromes. Parkinson's disease has a high prevalence in the elderly population, and its symptoms, such as movement disorders and cognitive decline, often overlap with the debilitating, cognitively impaired manifestations of geriatric syndromes. Therefore, although Parkinson's disease is a neurodegenerative disease in terms of medical classification, it is closely related to geriatric syndromes from the point of view of clinical practice, and together they affect the quality of life of the elderly. Depression is an affective disorder characterized by persistent low mood. Severe cases may include slowness of thought and behavior, as well as various somatization symptoms 7. The WHO predicts that by 2030, depression will become the leading cause of disease burden 8. Given the significant health, economic, and social burdens imposed by these conditions, there is an urgent need for a comprehensive understanding of their underlying mechanisms and appropriate treatment.

The gut microbiota constitutes a vital component of the human microecosystem. Microbiome alterations are acknowledged among the hallmarks of aging 9. Complex interactions between microbes and hosts during aging have been suggested to either accelerate or delay the onset of aging 10, providing a biomedical basis for preventing and treating age-related syndromes. However, the causal relationship between senescence and commensal microbes remains unclear.

Recently, there has been increased investigation into the relationship between gut microbiota and the health of older adults. Research has shown that changes in the microbiota are influenced by factors such as age, polypharmacy, lifestyle, and diet. Older adults have altered gut microbiota structure and diversity compared to younger individuals, which can lead to various disorders 11. However, the specific mechanisms underlying the link between gut microbiota and frailty remain unknown and require further investigation. Frail older adults often experience significant dietary changes due to declines in hearing, vision, mobility, and chewing ability. Additionally, reduced physical activity and gut motility, as well as changes in the living environment due to decreased self-care ability, can all impact the composition of gut microorganisms. Many bioactive metabolites produced by the microbiome are known to accumulate with aging have been implicated in various aspects of frailty 9. Moreover, alterations in the microbiome may lead to a loss of control over the rate of accumulation of senescent cells, which could have a significant impact on frailty 12.

PD is significantly influenced by the gut-brain axis, which links gastrointestinal microbiota, neural development, and neurological diseases 13. Short-chain fatty acids (SCFAs) such as butyrate, propionate, isobutyrate, and valerate are reduced in the feces of PD patients, suggesting that early pathogenic pathways in the gut may play a role in PD development. These SCFAs can trigger immune responses in the brain, leading to inflammation, which may contribute to cell damage and the various symptoms of PD 14. Changes in the microbiota may lead to metabolic alterations in patients with PD, with SCFA being the most examined gut microbial metabolite. A potential hypothesis is that elevated SCFA levels triggered by PD pathogenesis may be a secondary trigger for the development of PD 15. Ecological dysregulation of the gut microbiota may be further exacerbated by secondary changes in SCFAs levels. Studies have shown that SCFAs have certain beneficial functions, such as protecting the integrity of the intestinal barrier 16 and reducing the permeability of the blood-brain barrier 17. Contrary views also exist based on *in vivo* and *in vitro* evidence 18, 19. Various studies have attempted to elucidate the mechanism of action of SCFAs in PD; however, there are still many unanswered questions, and the results of different studies may differ or even contradict each other 17, 20, 21, and further studies are needed to characterize the role of SCFAs in the pathogenesis of PD and the exact mechanism.

Insomnia is the most prevalent sleep condition, and mounting research indicates that gut bacteria may play a role in its etiology 22, 23. Previous research has demonstrated links between biological cycles, immunological response, and nutrient metabolism, all of which may contribute to the prevalence of insomnia 24-26. Furthermore, substantial evidence suggests that gut microbiota not only affects host digestion, metabolism, and immune function but can also regulate host sleep through the microbiota-gut-brain axis 25, 27. The exact etiology and pathogenesis of depression have remained elusive. A study led by Guillaume Méric, a Finnish microbial bioinformatician, analyzed the genetic makeup and gut microbiome status of over 6,000 subjects and concluded that certain gut microbes, such as Morganella and Klebsiella, are associated with depression, with the underlying transmission mechanism related to genes 28.

Older adults often present with multiple coexisting diseases, complex etiologies, and undergo polypharmacy interventions, making it challenging to conduct single-disease gut microbiota studies on this population. Consequently, establishing causal analyses becomes essential for a better understanding of the mechanisms originating from the gut microbiota and for providing new perspectives for microbiome-focused therapeutic approaches. Traditional observational epidemiological studies face limitations due to the potential for confounding and reverse causality. To address these limitations, Mendelian randomization (MR) is a valuable technique used to discern causal relationships between exposures and outcomes 29, 30. By utilizing genetic variants closely related to the exposure as instrumental variables (IV), MR serves as a robust method for determining causal links between exposures and outcomes. MR can be thought of as a natural randomized controlled trial (RCT), offering strong evidence and being less susceptible to confounding variables. In contrast to single-sample MR methods, two-sample MR (TSMR) is particularly effective and powerful for establishing associations between “genetic risk factors” and “genetic outcomes.” However, this approach has not been previously used to explore a causal relationship between gut microbiota and geriatric syndromes. To assess the relationship between gut microbiota composition and geriatric syndromes, we conducted comprehensive two-sample MR analyses for five disorders using data from the IEU Open GWAS project including frailty, PD, psychosis, insomnia, and depression.

## Methods

### Study design

**Figure 1:**
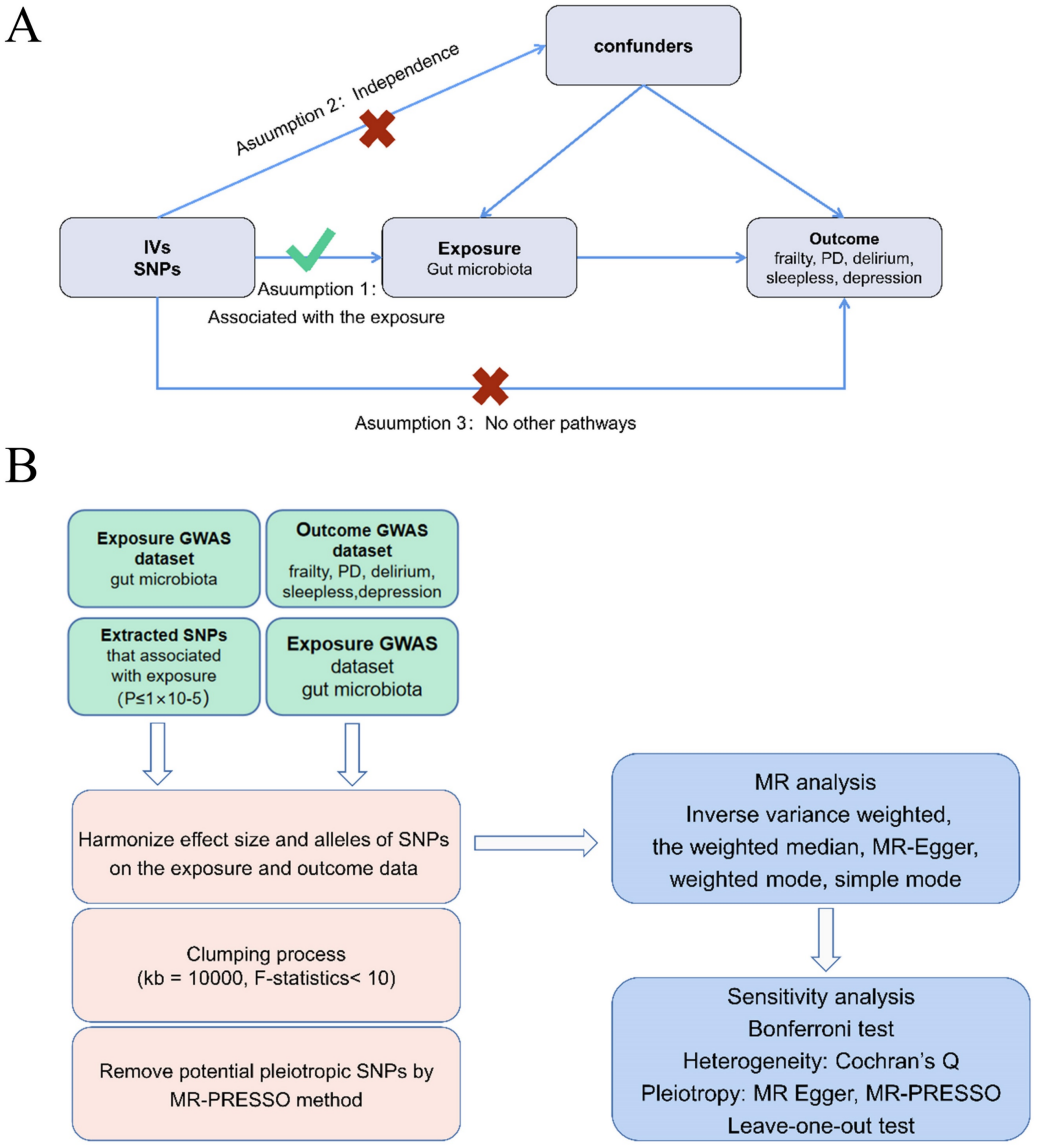
Flowchart describing the Mendelian randomization investigation in this study. MR, Mendelian randomization; PD, Parkinson's Disease (A) and three assumptions of Mendelian randomization (B).

Figure 1A presents a flowchart illustrating the MR analysis. Through the TSMR analysis, we identified a connection between several gut bacterial families and five prevalent geriatric syndrome disorders: frailty, PD, delirium, sleeplessness, and depression. In the initial step, where we considered the gut microbiome as the exposure and the five diseases as the outcomes, we aimed to determine whether the gut microbiota plays a role in either promoting or preventing these disorders.

To perform a two-sample MR, Bowden *et al.* outline three essential assumptions 31: (1) a strong correlation exists between SNPs and exposure factors; (2) confounding factors do not influence SNPs; (3) SNPs solely impact the outcomes through exposure factors (Figure 1B).

### Data from genome-wide association studies

We utilized SNPs linked to the human gut microbiota as instrumental variables (IVs) obtained from the MiBioGen Genome-Wide Association Study (GWAS) dataset, provided by the International Consortium (https://mibiogen.gcc.rug.nl/). MiBioGen collected samples from 18 populations and 19,000 individuals worldwide, including 16S rRNA sequencing data of the gut microbiota and genome-wide SNP data. They conducted a large-scale meta-GWAS analysis and have established a complete, open-source, and standardized analysis process. This process effectively eliminates technical errors caused by 16S rRNA amplification intervals 32.

For this study, we focused on five prevalent disorders in geriatric syndromes and summarized findings from publicly available GWAS analyses. The Frailty Index (FI) is a quantitative measure comprising more than 40 components and is reported as a ratio of the total number of age-related health deficiencies, serving as a continuous measure for assessing frailty. FI data were obtained from the IEU Open GWAS database (https://gwas.mrcieu.ac.uk), which includes data from 42,351 GWAS datasets.

Summary statistics for PD were sourced from the IEU Open GWAS database, which contains data from 449,056 European-ancestry controls and 33,674 cases 33. These statistics were based on the seventh iteration of the Finngen Biobank, a prospective cohort study with 342,499 participants as of December 2022 34. Summary statistics for insomnia and depression were obtained from the UK Biobank study. The UK Biobank produced GWAS summary statistics on insomnia and depression based on data from 501,500 and 122,938 United Kingdom residents, respectively (https://www.ukbiobank.ac.uk/).

### Choosing genetic manipulative variables

In this study, the gut microbiome served as the exposure, and we investigated its potential causal relationship with five common disorders in geriatric syndromes. To ensure the accuracy and reliability of the results, we implemented several control procedures. Firstly, we selected SNPs that showed significant associations with the gut microbiota as the IVs. To ensure the truth and accuracy of the causal relationship between gut microbes and diseases, we identified SNPs with *p*-values below the significance threshold of 1105 for further analysis 35.

In addition, we set the linkage disequilibrium coefficient R^2^<0.01 and the region width was set as 10,000 kb to exclude the effect of gene pleiotropy. F-statistics were used to estimate the strength of instrumental variables. Among them F<10, assuming a weak instrumental variable bias. SNPs with palindromic structures were automatically excluded during the analysis. SNPs with palindromic structures were automatically excluded. Thirdly, we applied a minor allele frequency (MAF) threshold of 0.01 to the variant of interest. This ensured that rare alleles were considered in our analysis 36.

To evaluate the potential effects of horizontal pleiotropy, we used two regression tests: namely MR-PRESSO and MR-Egger. MR-PRESSO helps exclude specific SNPs, eliminating outliers to obtain estimates closer to the true values. MR-Egger did not constrain the regression lines to pass through the origin, allowing for the presence of directed genetic pleiotropy among the instrumental variables. When the regression intercept is nonzero and *p* < 0.05, it indicates the presence of genetic pleiotropy.

To address potential pleiotropy, we sequentially removed each SNP from the list and retested the remaining SNPs globally using the MR-PRESSO test. The global test's p-value was iterated upon until it reached statistical significance (*p* > 0.05), at which point the process was repeated. The list of SNPs that remained after accounting for pleiotropic effects was used for the MR analysis to ensure the accuracy of our findings.

### Statistical analysis

We used a two-sample MR method to investigate the potential relationship between the composition of the gut microbiota and the presence of frailty. To explore potential causal connections between the gut microbiota and frailty, we used five distinct MR techniques, including the inverse variance weighted (IVW) method 37, the weighted median 38, MR-Egger 31, the weighted mode method 39, and the simple mode. The IVW approach served as the primary analysis method to provide precise estimations, with the other four methods used as supplementary analyses 40. Moreover, we assessed potential horizontal pleiotropy effects using MR-PRESSO and MR-Egger.

For sensitivity analysis, we employed the “leave-one-out” approach from the R package. This involved systematically reanalyzing the results by sequentially removing each instrumental variable (IV) to evaluate the influence of each SNP on the outcome. The results of this analysis were presented in a forest plot. To minimize the impact of measurement errors in the included IVs, we conducted a heterogeneity test. Cochran's Q test was used to evaluate potential bias in the causal effect estimates due to measurement errors stemming from diverse analysis platforms, experimental setups, and study populations. This test was calculated using the “mr_heterogeneity” function from the “TwoSampleMR” package. We considered heterogeneity not to affect the study results when the test result indicated *P* > 0.05 41. The results were presented in a table. Additionally, we used the Bonferroni correction to assess the significance of multiple testing at each feature level (*P* < 0.05/n, where n is the number of bacterial taxa included in each feature level) to more precisely identify causal associations 35.

## Results

### Instrumental variable selection

First, we identified 14,587 SNPs associated with the gut microbiota from the MiBioGen Consortium at a stringent significance level (*P*<1×10^-5^). Furthermore, none of the IV's had an F-statistic lower than 10, mitigating the potential for weak instrument bias. Specifically, 101 independent SNPs were associated with 7 microbiomes in frailty, 80 SNPs with 7 microbiomes in PD, 70 SNPs with 5 microbiomes in delirium, 143 SNPs with 11 microbiomes in insomnia, and 137 SNPs with 11 microbiomes in depression (Supplementary Tables).

### Causal effects of gut microbiota on frailty

**Figure 2:**
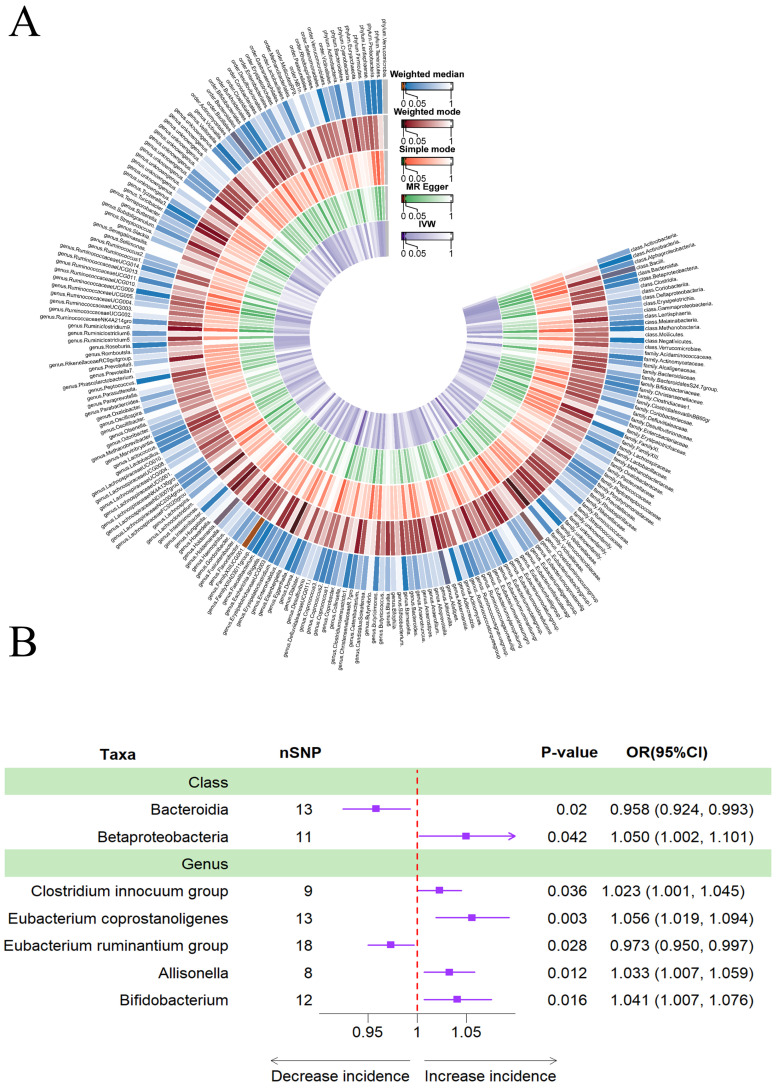
(A)Causal effects of the gut microbiome on frailty based on MR analyses. From inside to outside, the P values of IVW, MR Egger, SM, Wmode and WM represented, respectively. IVW, inverse variance weighted; SM, simple mode; Wmode weighted mode; WM, weighted median. (B)Forest plot showing Mendelian randomization results for causal effects of gut microbiota on frailty risk. CI: confidence interval; OR: odds ratio.

Figure 2A provided evidence of the causal effects of 196 gut microbiomes on the occurrence of frailty. According to Table 1, a lower risk of frailty was associated with a higher genetically predicted abundance of class *Bacteroidia* (OR: 0.958, 95% CI: 0.924-0.993, *p* = 0.020) and genus *Eubacterium ruminantium* group (OR: 0.973, 95% CI: 0.950-0.997, *p* = 0.028). In contrast, class *Betaproteobacteria* (OR: 1.050, 95% CI: 1.002-1.101, *p* = 0.042), genus *Clostridium innocuum* group (OR: 1.023, 95% CI: 1.001-1.045, *p* = 0.036), genus *Eubacterium coprostanoligenes* (OR: 1.056, 95% CI: 1.019-1.094, *p* = 0.003), genus *Allisonella* (OR: 1.033, 95% CI: 1.007-1.059, *p* = 0.012) and genus *Bifidobacterium* (OR: 1.041, 95% CI: 1.007-1.076, *p* = 0.016) showed a positive genetic relationship with frailty risk (Figure 2B).

### Causal effects of gut microbiota on PD

**Figure 3:**
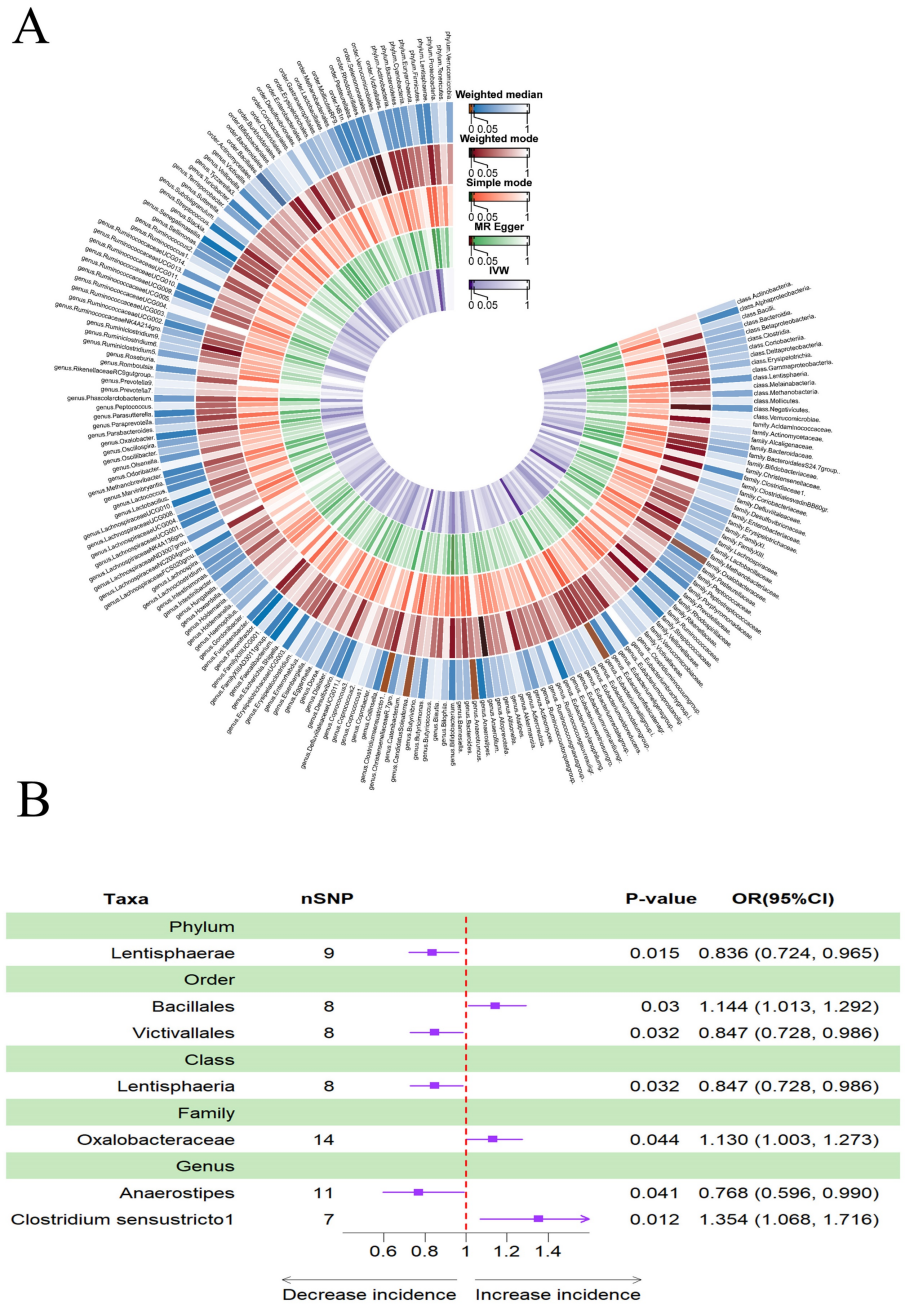
(A)Causal effects of the gut microbiome on Parkinson's disease (PD) based on MR analyses. From inside to outside, the P values of IVW, MR Egger, SM, Wmode and WM represented, respectively. IVW, inverse variance weighted; SM, simple mode; Wmode weighted mode; WM, weighted median. (B)Forest plot of Mendelian randomization results for causal effects of gut microbiota on PD risk.

In addition, Figure 3A provided evidence of the causal effects of 196 gut microbiomes on the occurrence of PD. The results obtained using the IVW method revealed that a lower risk of PD was associated with a higher genetically predicted abundance of phylum *Lentisphaerae* (OR: 0.836, 95% CI: 0.724-0.965, *p* = 0.015), order *Victivallales* (OR: 0.847, 95% CI: 0.728-0.986), class *Lentisphaeria* (OR: 0.847, 95% CI: 0.728-0.986), and genus *Anaerostipes* (OR: 0.768, 95% CI: 0.596-0.990, *p* = 0.041). Conversely, the genetically predicted abundance of the family *Oxalobacteraceae* (OR: 1.130, 95% CI: 1.003-1.273, *p* = 0.044), genus *Clostridium sensu stricto1* (OR: 1.354, 95% CI: 1.068-1.716, *p* = 0.012), and order *Bacillales* (OR: 1.144, 95% CI: 1.013-1.292, *p* = 0.030) showed a positive correlation with the risk of PD (Table 2, Figure 3B).

### Causal effects of gut microbiota on delirium

**Figure 4:**
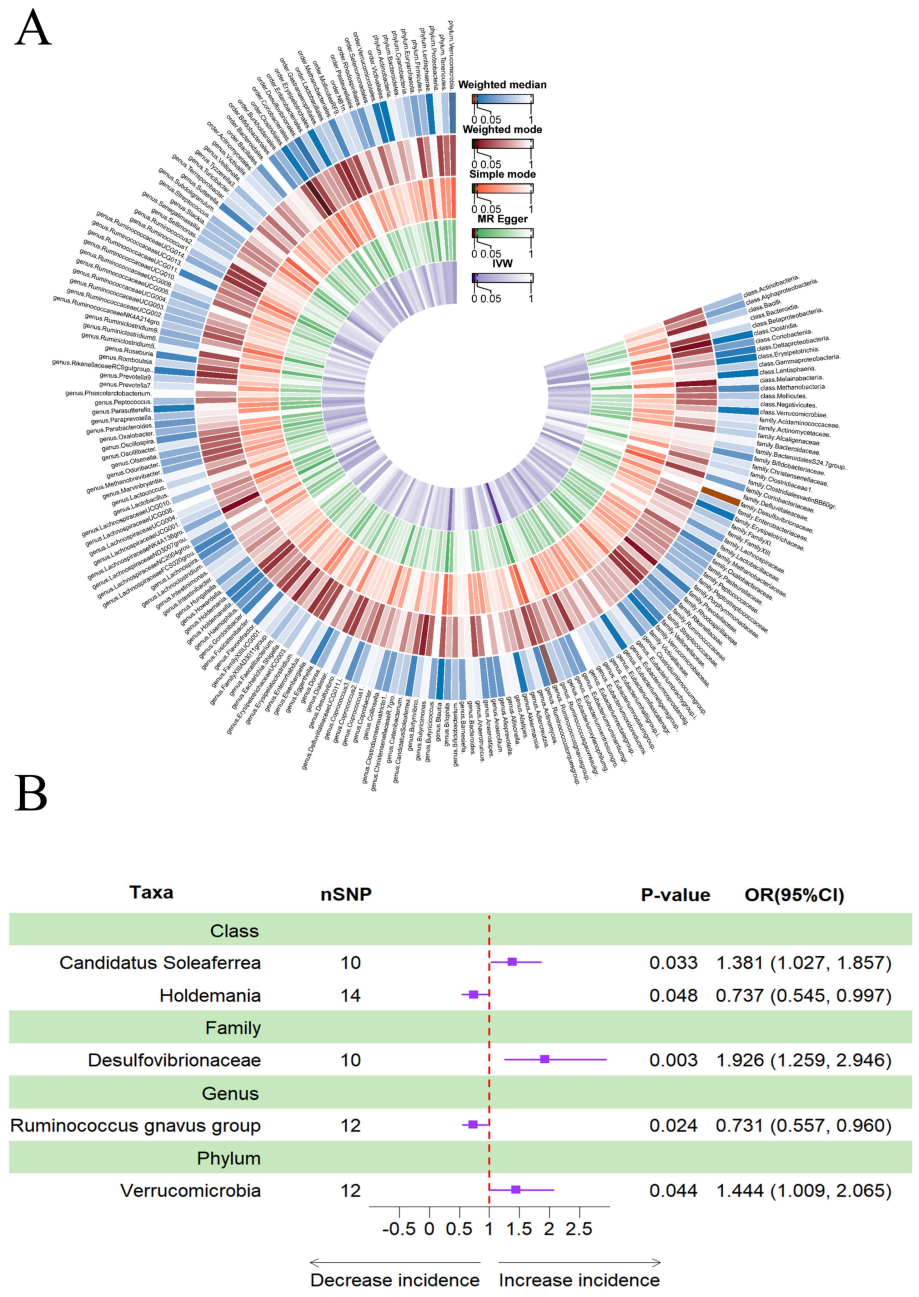
(A) Causal effects of the gut microbiome on delirium based on MR analyses. From inside to outside, the P values of IVW, MR Egger, SM, Wmode and WM represented, respectively. IVW, inverse variance weighted; SM, simple mode; Wmode weighted mode; WM, weighted median. (B) Forest plot of Mendelian randomization results for causal effects of gut microbiota on delirium risk.

Figure 4A provided evidence of the causal effects of 196 gut microbiomes on the occurrence of delirium. The IVW method revealed that a lower risk of delirium was associated with a higher genetically predicted abundance of the genus *Ruminococcus gnavus* group (OR: 0.731, 95% CI: 0.557-0.960, *p* = 0.024) and class *Holdemania* (OR: 0.737, 95% CI: 0.737-0.545, 0.997, *p* = 0.048) (Table 3). Conversely, phylum *Verrucomicrobia* (OR: 1.444, 95% CI: 1.009-2.065, *p* = 0.044), family *Desulfovibrionaceae* (OR: 1.926, 95% CI: 1.259-2.946, *p* = 0.003) and class *Candidatus Soleaferrea* (OR: 1.381, 95% CI: 1.027-1.857, *p* = 0.033) showed a positive genetic relationship with delirium risk (Figure 4B).

### Causal effects of gut microbiota on insomnia

**Figure 5:**
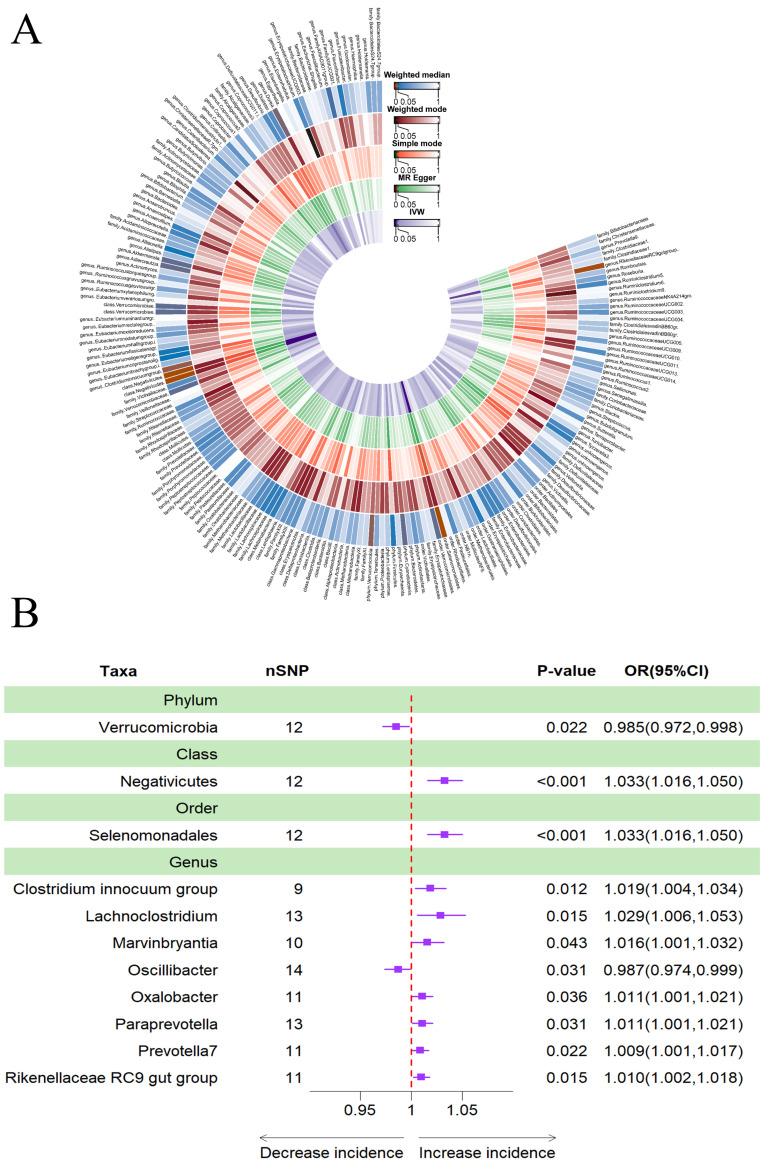
(A) Causal effects of the gut microbiome on insomnia based on MR analyses. From inside to outside, the P values of IVW, MR Egger, SM , Wmode and WM represented, respectively. IVW, inverse variance weighted; SM, simple mode; Wmode weighted mode; WM, weighted median. (B) Forest plot of Mendelian randomization results for causal effects of gut microbiota on insomnia risk.

The results obtained using the IVW method indicated that a higher genetically predicted abundance of phylum *Verrucomicrobia* (OR: 0.985, 95% CI: 0.972**-**0.998, *p* = 0.022) and genus *Oscillibacter* (OR: 0.987, 95% CI: 0.974**-**0.999, *p* = 0.031) was associated with a reduced risk of sleeplessness (Table 4). In contrast, class *Negativicutes* (OR: 1.033, 95% CI: 1.016**-**1.050, *p* = 0.000), order *Selenomonadales* (OR: 1.033, 95% CI: 1.016-1.050, *p* = 0.000), genus *Clostridium innocuum* group (OR: 1.019, 95% CI: 1.004**-**1.034, *p* = 0.012), genus *Lachnoclostridium* (OR: 1.029, 95% CI: 1.006**-**1.053, *p* = 0.015), genus *Marvinbryantia* (OR: 1.016, 95% CI: 1.001**-**1.032, *p* = 0.043), genus *Oxalobacter* (OR: 1.011, 95% CI: 1.001**-**1.021, *p* = 0.036), genus *Paraprevotella* (OR: 1.011, 95% CI: 1.001**-**1.021, *p* = 0.031), genus *Prevotella7* (OR: 1.009, 95% CI: 1.001**-**1.017, *p* = 0.022) and genus *Rikenellaceae RC9* gut group (OR: 1.010, 95% CI: 1.002**-**1.018, *p* = 0.015) showed a positive genetic relationship with the risk of insomnia (Figure 5B). Additionally, Figure 5A provided evidence of the causal effects of 196 gut microbiomes on the occurrence of insomnia.

### Causal effects of gut microbiota on depression

**Figure 6:**
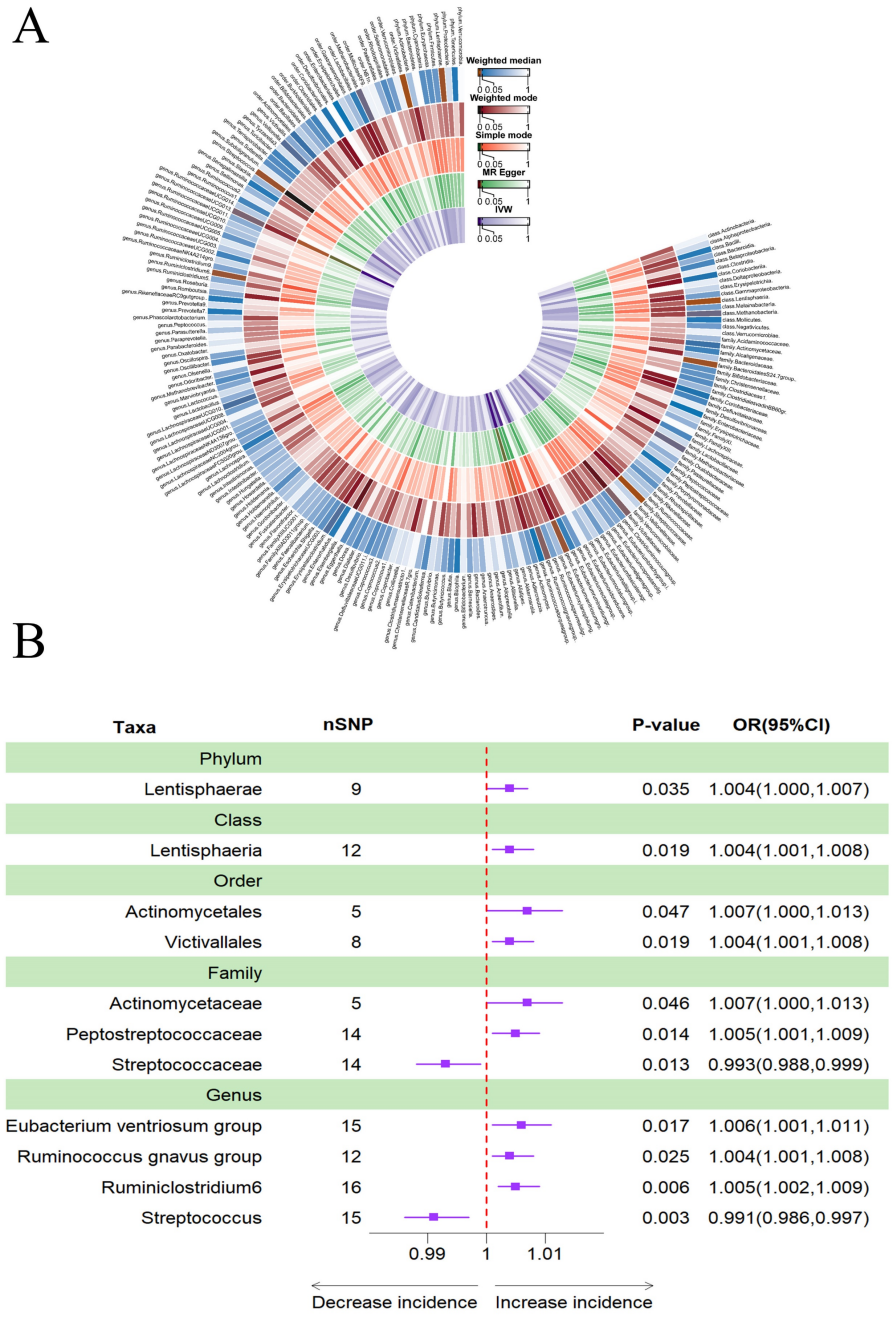
(A) Causal effects of the gut microbiome on depression based on MR analyses. From inside to outside, the P values of IVW, MR Egger, SM, Wmode and WM represented, respectively. IVW, inverse variance weighted; SM, simple mode; Wmode weighted mode; WM, weighted median. (B) Forest plot of Mendelian randomization results for causal effects of gut microbiota on depression risk.

According to Table 5, a higher genetically predicted abundance of phylum *Lentisphaerae* (OR: 1.004, 95% CI: 1.000-1.007, *p* = 0.035), class *Lentisphaeria* (OR: 1.004, 95% CI: 1.001-1.008, *p* = 0.019), order *Actinomycetales* (OR: 1.007, 95% CI: 1.000-1.013, *p* = 0.047), order *Victivallales* (OR: 1.004, 95% CI: 1.001-1.008, *p* = 0.019), family *Actinomycetaceae* (OR: 1.007, 95% CI: 1.000-1.013, *p* = 0.046), family *Peptostreptococcaceae* (OR: 1.005, 95% CI: 1.001-1.009, *p* = 0.014), genus *Eubacterium ventriosum* group(OR: 1.006, 95% CI: 1.001-1.011, *p* = 0.017), genus *Ruminococcus gnavus* group (OR: 1.004, 95% CI: 1.001-1.008, *p* = 0.025), and genus *Ruminiclostridium6* (OR: 1.005, 95% CI: 1.002-1.009, *p* = 0.006) were associated with a reduced risk of depression. Conversely, family *Streptococcaceae* (OR: 0.993, 95% CI: 0.988-0.999, *p* = 0.013) and genus *Streptococcus* (OR: 0.991, 95% CI: 0.986-0.997, *p* = 0.003) showed a positive genetic relationship with the risk of depression (Figure 6B). Figure 6A provided evidence of the causal effects of 196 gut microbiomes on the occurrence of depression.

## Discussion

As far as we are aware, our two-sample MR study is the first attempt to use a publicly accessible genetic database to investigate the causal link between the gut microbiota and five geriatric disorders: frailty, PD, delirium, insomnia, and depression. Our research demonstrates that 41 gut microbiota are causally linked to geriatric syndromes and their phenotypes, significantly advancing our understanding of the gut microbiota's role in the pathology of these conditions. These findings provide fresh insights into prevention and diagnosis strategies for these conditions.

There is mounting evidence that the so-called “gut-brain axis” influences the risk of several age-related chronic diseases and syndromes, including frailty and neurodegenerative diseases 42. Age-related frailty is a distinctive geriatric syndrome characterized by lower gut microbial diversity in the older adults compared to younger individuals, with significant interindividual variations.

Previous research has suggested that a low distinctness index of the gut microbiome and a high prevalence of *Bacteroides* are independently associated with mortality in older adults. Conversely, a high abundance of *Lactobacillus* and *Bifidobacterium* is indicative of a healthier microbiome, often observed in centenarians 43.

Our findings indicate a correlation between a higher genetic abundance of *Bacteroidia* and frailty, suggesting an increase in *Bacteroides* in frail older adults 44. *Bifidobacteria* have also been recognized as an important factor in sarcopenia and frailty among older adults 45. Intestinal microbiota associated with frailty and sarcopenia have been linked to changes in the abundance of *Bifidobacterium,* which is associated with better health, as suggested by animal studies. *Bifidobacterium* has shown the potential to significantly reduce the peripheral tiredness index associated with exercise in mice and decrease the damage index associated with oxidative stress, possibly through its role in regulating inflammation 10, 46. Both the MR Egger and weighted median techniques consistently revealed directional effects across all studies, suggesting that *Bacteroidia* may be a promising target for frailty prevention.

In frail older adults, *Eubacterium* is less prevalent 47-49. Due to its production of short-chain fatty acids (SCFAs), particularly butyrate, and its role in immune system regulation, *Eubacterium* is considered a protective colonic bacterium 50. Abundance of *Eubacterium* is inversely correlated with gut health, and its decline may have systemic consequences. The reduction of *Eubacterium* in older adults and frail individuals may contribute to the protective impact of SCFAs on the human gut 49.

However, further research is needed to elucidate the precise mechanism. It remains unclear how the other bacterial genera in our study, which showed significant alterations, may be associated with the onset of frailty.

Our findings demonstrated a potential causal relationship between the increased diversity of the phylum *Lentisphaerae*, class *Lentisphaeria*, and order *Victivallales* and a potential protective effect against PD. However, as this observation did not survive multiple corrections and has not been reported in previous literature, a definitive conclusion about causality cannot be drawn. It should be interpreted with caution and seen as a potential causal relationship.

The associations between family *Oxalobacteraceae*, order *Bacillales*, *Anaerostipes*, and *Clostridium sensu stricto* 1 and PD are consistent with previous results 21, 51, 52. Furthermore, our investigation revealed that several microbial clusters capable of producing SCFAs, including *Clostridium sensu stricto* 1 and *Bacillales*, were associated with a higher risk of PD 53. Increasing SCFA levels are generally considered beneficial for health 54, and PD symptoms can be mitigated or even eliminated by introducing SCFAs or reestablishing gut flora 55, 56.

The exact mechanism by which gut microbial dysbiosis contributes to PD remains unclear. Microbial SCFAs could potentially be one of the primary mediators of the microbiota-gut-brain axis, with a role in the onset and progression of PD. To uncover the function and precise mechanisms of SCFAs in the pathogenesis of PD, further research will be necessary in the future.

Data on gut microbiota dysbiosis in acute neuropsychiatric illnesses are currently lacking. Delirium, characterized by inattention, disorganized thinking, and altered awareness that fluctuates over time, is the most prevalent acute neuropsychiatric problem in hospitalized older adults. Delirium is associated with a range of negative outcomes, including functional and cognitive decline, the need for hospitalization, and increased mortality 57.

One study found that an animal model of postoperative delirium showed a dysbiotic gut microbiome, with decreased levels of *Ruminococcus* and *Roseburia* and increased levels of *Rikenellaceae* in fecal samples of postoperative delirious mice. These findings are similar to our results, although the exact mechanisms remain unclear 58. Notably, postoperative delirious patients had high levels of *Proteobacteria, Enterobacteriaceae, Escherichia shigella, Klebsiella, Ruminococcus, Roseburia, Blautia, Holdemanella, Anaerostipes, Burkholderiaceae, Peptococcus, Lactobacillus*, and *Dorea,* whereas patients without postoperative delirium had high levels of *Streptococcus* 59. This research provides new perspectives and approaches for the prevention and treatment of delirium, which is crucial for improving the well-being and quality of life of older adults. However, further research is needed to determine how these bacteria may be related to the potential causes of postoperative delirium.

Currently, there is evidence suggesting a certain association between gut microbiota and insomnia. Moreover, *Firmicutes* and *Proteobacteria* were more abundant in healthy individuals compared to insomnia patients, leading to a reduced *Firmicutes*-to-*Bacteroidetes* ratio, with *Bacteroidetes* being the predominant phylum in the insomnia group 60. Moreover, insomnia patients showed a significant decrease in the genus *Bacteroides* and a notable increase in the genus *Prevotella*. They also showed a prevalence of *Gemmiger* and *Fusicatenibacter*. In contrast, *Peptostreptococcaceae, Coprococcus, Oscillibacter*, and the genus *Clostridium* were dominant in healthy individuals 24. Moreover, a strong correlation was observed between higher sleep efficiency and cognitive ability and the presence of the genus *Lachnoclostridium* 61, aligning with our findings. Research by Agrawal *et al.* 62 indicated that short sleepers had a lower relative abundance of *Lactobacillus* compared to regular sleepers.

This study identified a strong correlation between a higher abundance of the class *Negativicutes* and order *Selenomonadales* and an increased incidence of sleeplessness 63. Notably, melatonin is a common treatment for improving sleep in individuals with insomnia and can also address gut microbiota issues arising from sleep disturbances. Studies involving melatonin treatment in sucking piglets demonstrated a reduction in the prevalence of order *Selenomonadales* 64. These findings suggest that by promoting the operation of gut neural networks and the gut barrier, the order *Selenomonadales* and class *Negativicutes* class may considerably increase the efficiency of melatonin in treating the symptoms of insomnia 64.

Moreover, this study found a potential association between the genera of *Marvinbryantia, Oxalobacter, Paraprevotella*, and *Rikenellaceae* RC9 gut group, with insomnia. This finding represents a novel discovery as it has not been previously reported in existing studies. However, it is needed to do further study to validate and confirm this association.

In previous studies, many researchers have conducted extensive research linking gut microbiota to depression from various perspectives and through different experimental methods 65-67. However, the contribution of family *Streptococcaceae* and genus *Streptococcus* to the pathophysiology of depression remains unclear, as there are limited experimental reports on this topic, warranting further investigation.

According to a case-control study, pro-inflammatory genera like *Streptococcus* were enriched, while anti-inflammatory genera like *Faecalibacterium* were decreased in the depressed group 68. Additionally, our MR study identified a potential association between depression and a higher prevalence of *Streptococcaceae* or *Streptococcus.* Furthermore, the genus *Ruminococcus gnavus* has been linked to mental and behavioral issues in children, including withdrawal, anxiety, despair, and muscle soreness 69. *Ruminococcus* has also been associated with various psychiatric conditions, including schizophrenia, mood disorders, and major depressive disorder 70-72. *Ruminococcus* plays a role in metabolic processes involving the breakdown of mucin and complex sugars, both of which are essential for providing additional energy 73.

Moreover, *Ruminococcus* metabolites, particularly SCFAs, are significant chemicals that influence human behavior and brain function, which may contribute to depression 74. *Ruminococcus* has the potential to impact lipids, including phosphoethanolamine and glycerophosphorylcholine as well as inflammatory signaling pathways, including the NLRP3 inflammasome, which may contribute to the etiology of depression 75. However, further research is needed to validate this hypothesis. In conclusion, the mechanisms through which *Streptococcus* and *Ruminococcus* influence depression warrant continued exploration in future studies.

Our study has several advantages. Firstly, this is the first MR investigation that has assessed the causal relationship between geriatric illnesses and gut microbiota. This approach reduces the susceptibility to confounding and reverse causality compared to traditional observational studies when examining the connection between gut microbiota and five geriatric syndromes. However, the potential influence of horizontal pleiotropy cannot be completely eliminated due to the uncertain biological mechanisms of many genetic variants. Therefore, the findings should be interpreted with caution. Secondly, by investigating the causative relationships between diverse gut microbiota, from genus to phylum, and diseases, we gained new insights into how to target specific gut bacteria in clinical practice to prevent and treat geriatric syndromes. Thirdly, the reliability and robustness of the causal links indicated by the MR investigation were improved by rigorous quality control procedures and the use of multiple sensitivity analysis techniques. Nonetheless, this study has certain limitations. First, Mendelian randomization relies on the assumption of exclusivity that genetic variation as an instrumental variable affects the outcome only through the exposure factor of interest. Although confounding factors or multiple effects at the gene level were avoided as much as possible, and MR-Egger regression method and MRPRESSO method were used to further ensure the stability of the study results, unmeasured confounding factors may still exist. For example, medications are a potential confounder affecting the composition of the gut microbiota. Antibiotics lead to the reduction of gut microbial diversity and imbalance of gut microbiota by killing or inhibiting specific bacterial groups, a phenomenon commonly referred to as' dysbiosis' 76. Previous studies have shown that antibiotic use is associated with multiple long-term health outcomes, including an increased risk of frailty 77. In addition, dietary habits are another important factor in shaping the structure of the gut microbiota, which has a direct impact on the microbial fermentation process by providing different types and amounts of substrates 78. Thus, although our study provides valuable insights into the relationship between gut microbiota and frailty, these findings still need to be further validated in future studies with stricter control for confounders and more precise exposure assessment. It is worth mentioning that while the issue of pleiotropy may never be proved, it is generally accepted. However, it can be verified that pleiotropy has no effect on the results by performing sensitivity analyses of various MR Models based on different assumptions and methods, complementing each other and corroborating each other. Second, this study only included European populations, and the generalizability of the results may be limited. Therefore, further research in other populations is required. Third, the GWAS data for gut microbiota used in this study was based on the largest population cohort ever analyzed through metagenomic sequencing. However, to comprehensively assess the causal association between gut microbiota and geriatric disorders, summary data from additional gut microbiota will be necessary in the future. Additionally, this study could not to calculate the overlap between participants in exposure and outcome GWASs, which might lead to an overestimation of the study results. Furthermore, the investigation was unable to establish reverse causation due to limited availability of instrumental variables (IVs).

## Conclusions

In conclusion, this study provided a comprehensive assessment of the causal relationship between gut microbiota and geriatric syndromes. Using two-sample MR analyses, we identified associations between 7 gut microbiota and frailty, 7 with PD, 5 with delirium, 11 with insomnia, and 11 with depression. It's important to note that the causal relationships derived from MR analyses represent statistical causal relationships and not exact causation. However, our work does present evidence of potential causal links between specific gut microbiota and five geriatric syndromes. These findings contribute to a better understanding of the potential pathways through which gut microbiota may influence geriatric syndromes. Nevertheless, larger GWAS data and further validation through additional MR studies will be in the future to confirm and expand upon these findings.

## Figures and Tables

**Table 1:** Mendelian randomization result of casual effects between gut microbiome and the risk of frailty.

Group	Bacterial	Nsnp	Methods	SE	OR (95% CI)	P-value
Class	Bacteroidia	13	Inverse variance weighted	0.018	0.958 (0.924, 0.993)	0.020
			MR Egger	0.042	0.944 (0.870, 1.026)	0.202
			Simple mode	0.039	0.954 (0.885, 1.030)	0.257
			Weighted median	0.026	0.960 (0.913, 1.009)	0.107
			Weighted mode	0.031	0.957 (0.901, 1.016)	0.174
	Betaproteobacteria	11	Inverse variance weighted	0.024	1.050 (1.002, 1.101)	0.042
			MR Egger	0.087	1.021 (0.861, 1.210)	0.820
			Simple mode	0.048	1.092 (0.993, 1.200)	0.100
			Weighted median	0.028	1.079 (1.022, 1.139)	0.006
			Weighted mode	0.048	1.087 (0.990, 1.194)	0.112
Genus	Clostridiuminnocuum group	9	Inverse variance weighted	0.011	1.023 (1.001, 1.045)	0.036
			MR Egger	0.055	1.133 (1.017, 1.263)	0.059
			Simple mode	0.023	1.017 (0.972, 1.065)	0.490
			Weighted median	0.015	1.022 (0.993, 1.052)	0.137
			Weighted mode	0.022	1.017 (0.974, 1.062)	0.465
	Eubacterium coprostanoligenes	13	Inverse variance weighted	0.018	1.056 (1.019, 1.094)	0.003
			MR Egger	0.073	1.072 (0.929, 1.237)	0.363
			Simple mode	0.037	1.084 (1.008, 1.166)	0.051
			Weighted median	0.024	1.070 (1.021, 1.122)	0.005
			Weighted mode	0.037	1.085 (1.008, 1.168)	0.050
	Eubacteriumruminantiumgroup	18	Inverse variance weighted	0.012	0.973 (0.950, 0.997)	0.028
			MR Egger	0.043	1.042 (0.958, 1.133)	0.350
			Simple mode	0.025	1.003 (0.954, 1.054)	0.921
			Weighted median	0.015	0.997 (0.969, 1.027)	0.860
			Weighted mode	0.023	1.003 (0.959, 1.050)	0.891
	Allisonella	8	Inverse variance weighted	0.013	1.033 (1.007, 1.059)	0.012
			MR Egger	0.072	0.898 (0.779, 1.034)	0.186
			Simple mode	0.029	1.061 (1.003, 1.122)	0.078
			Weighted median	0.015	1.017 (0.988, 1.047)	0.255
			Weighted mode	0.022	1.003 (0.961, 1.046)	0.904
	Bifidobacterium	12	Inverse variance weighted	0.017	1.041 (1.007, 1.076)	0.016
			MR Egger	0.045	1.089 (0.997, 1.189)	0.089
			Simple mode	0.041	1.015 (0.935, 1.101)	0.732
			Weighted median	0.023	1.032 (0.987, 1.079)	0.166
			Weighted mode	0.042	1.014 (0.933, 1.101)	0.757

**Table 2:** MR result of casual effects between gut microbiome and the risk of PD.

Group	Bacterial	Nsnp	Methods	SE	OR (95% CI)	P-value
Phylum	Lentisphaerae	9	Inverse variance weighted	0.074	0.836 (0.724, 0.965)	0.015
			MR Egger	0.258	0.715 (0.431, 1.186)	0.235
			Simple mode	0.162	0.743 (0.542, 1.020)	0.104
			Weighted median	0.099	0.762 (0.628, 0.924)	0.006
			Weighted mode	0.149	0.745 (0.556, 0.998)	0.084
Order	Bacillales	8	Inverse variance weighted	0.077	1,144 (1.013, 1.292)	0.030
			MR Egger	0.255	0.743 (0.450, 1.225)	0.288
			Simple mode	0.166	0.747 (0.540, 1.035)	0.123
			Weighted median	0.105	0.783 (0.638, 0.962)	0.020
			Weighted mode	0.155	0.751 (0.555, 1.017)	0.107
	Victivallales	8	Inverse variance weighted	0.077	0.847 (0.728, 0.986)	0.032
			MR Egger	0.255	0.743 (0.450, 1.225)	0.288
			Simple mode	0.169	0.747 (0.537, 1.040)	0.128
			Weighted median	0.108	0.783 (0.634, 0.969)	0.024
			Weighted mode	0.163	0.751 (0.546, 1.033)	0.122
Class	Lentisphaeria	8	Inverse variance weighted	0.077	0.847 (0.728, 0.986)	0.032
			MR Egger	0.255	0.743 (0.450, 1.225)	0.288
			Simple mode	0.177	0.747 (0.528, 1.058)	0.145
			Weighted median	0.107	0.783 (0.635, 0.966)	0.022
			Weighted mode	0.160	0.751 (0.549, 1.027)	0.116
Family	Oxalobacteraceae	14	Inverse variance weighted	0.061	1.130 (1.003, 1.273)	0.044
			MR Egger	0.259	1.422 (0.856, 2.362)	0.198
			Simple mode	0.135	1.194 (0.915, 1.557)	0.213
			Weighted median	0.079	1.177 (1.008, 1.375)	0.040
			Weighted mode	0.137	1.202 (0.919, 1.571)	0.202
Genus	Anaerostipes	11	Inverse variance weighted	0.129	0.768 (0.596, 0.990)	0.041
			MR Egger	0.411	0.568 (0.254, 1.270)	0.201
			Simple mode	0.291	0.976 (0.552, 1.726)	0.935
			Weighted median	0.169	0.792 (0.569, 1.103)	0.168
			Weighted mode	0.304	1.024 (0.565, 1.858)	0.938
	Clostridium sensustricto1	7	Inverse variance weighted	0.121	1.354 (1.068, 1.716)	0.012
			MR Egger	0.275	1.728 (1.009, 2.959)	0.103
			Simple mode	0.220	1.404 (0.912, 2.161)	0.174
			Weighted median	0.160	1.413 (1.034, 1.933)	0.030
			Weighted mode	0.195	1.416 (0.966, 2.075)	0.125

**Table 3:** MR result of casual effects between gut microbiome and the risk of delirium.

Group	Bacterial	Nsnp	Methods	SE	OR (95% CI)	P-value
Phylum	Verrucomicrobia	12	Inverse variance weighted	0.183	1.444 (1.009, 2.065)	0.044
			MR Egger	0.478	1.740 (0.682, 4.438)	0.273
			Simple mode	0.411	2.053 (0.917, 4.596)	0.108
			Weighted median	0.259	1.505 (0.906, 2.500)	0.114
			Weighted mode	0.347	1.473 (0.746, 2.906)	0.288
Family	Desulfovibrionaceae	10	Inverse variance weighted	0.217	1.926 (1.259, 2.946)	0.003
			MR Egger	0.507	0.975 (0.361, 2.631)	0.961
			Simple mode	0.536	1.540 (0.538, 4.404)	0.441
			Weighted median	0.311	1.492 (0.811, 2.745)	0.198
			Weighted mode	0.351	1.252 (0.630, 2.490)	0.537
Genus	Ruminococcus gnavus group	12	Inverse variance weighted	0.139	0.731 (0.557, 0.960)	0.024
			MR Egger	0.656	0.538 (0.149, 1.943)	0.366
			Simple mode	0.269	0.608 (0.359, 1.030)	0.091
			Weighted median	0.187	0.648 (0.449, 0.933)	0.020
			Weighted mode	0.268	0.616 (0.365, 1.043)	0.099
Class	Candidatus Soleaferrea	10	Inverse variance weighted	0.151	1.381 (1.027, 1.857)	0.033
			MR Egger	1.621	1.344 (0.056, 32.211)	0.860
			Simple mode	0.343	1.132 (0.577, 2.219)	0.726
			Weighted median	0.201	1.240 (0.835, 1.840)	0.286
			Weighted mode	0.323	1.114 (0.592, 2.096)	0.747
	Holdemania	14	Inverse variance weighted	0.154	0.737 (0.545, 0.997)	0.048
			MR Egger	0.453	0.401 (0.165, 0.973)	0.066
			Simple mode	0.315	0.764 (0.412, 1.415)	0.407
			Weighted median	0.202	0.749 (0.504, 1.113)	0.153
			Weighted mode	0.319	0.738 (0.394, 1.380)	0.358

**Table 4:** Mendelian randomization result of casual effects between gut microbiome and the risk of insomnia.

Group	Bacterial	Nsnp	Methods	SE	OR (95% CI)	P-value
Phylum	Verrucomicrobia	12	Inverse variance weighted	0.007	0.985(0.972,0.998)	0.022
			MR Egger	0.019	0.983(0.947,1.020)	0.377
			Simple mode	0.015	0.990(0.961,1.019)	0.515
			Weighted median	0.009	0.986(0.969,1.003)	0.107
			Weighted mode	0.014	0.989(0.962,1.017)	0.450
Class	Negativicutes	12	Inverse variance weighted	0.008	1.033(1.016,1.050)	0.000
			MR Egger	0.026	1.042(0.990,1.098)	0.147
			Simple mode	0.019	1.047(1.008,1.087)	0.035
			Weighted median	0.011	1.046(1.023,1.069)	0.000
			Weighted mode	0.020	1.047(1.008,1.088)	0.038
Order	Selenomonadales	12	Inverse variance weighted	0.008	1.033(1.016,1.050)	0.000
			MR Egger	0.026	1.042(0.990,1.098)	0.147
			Simple mode	0.019	1.047(1.008,1.088)	0.038
			Weighted median	0.011	1.046(1.023,1.069)	0.000
			Weighted mode	0.020	1.047(1.008,1.088)	0.039
Genus	Clostridium innocuum group	9	Inverse variance weighted	0.007	1.019(1.004,1.034)	0.012
			MR Egger	0.040	0.998(0.924,1.079)	0.968
			Simple mode	0.009	1.008(0.990,1.027)	0.403
			Weighted median	0.007	1.012(0.997,1.026)	0.111
			Weighted mode	0.009	1.007(0.989,1.026)	0.448
	Lachnoclostridium	13	Inverse variance weighted	0.012	1.029(1.006,1.053)	0.015
			MR Egger	0.043	1.009(0.926,1.098)	0.844
			Simple mode	0.022	1.027(0.983,1.072)	0.254
			Weighted median	0.013	1.028(1.002,1.056)	0.038
			Weighted mode	0.022	1.024(0.980,1.070)	0.305
	Marvinbryantia	10	Inverse variance weighted	0.008	1.016(1.001,1.032)	0.043
			MR Egger	0.030	0.982(0.926,1.043)	0.576
			Simple mode	0.020	1.028(0.988,1.069)	0.207
			Weighted median	0.010	1.0182(0.998,1.039)	0.078
			Weighted mode	0.020	1.026(0.986,1.067)	0.233
	Oscillibacter	14	Inverse variance weighted	0.006	0.987(0.974,0.999)	0.031
			MR Egger	0.023	1.007(0.962,1.053)	0.779
			Simple mode	0.014	0.983(0.957,1.011)	0.250
			Weighted median	0.008	0.987(0.972,1.003)	0.109
			Weighted mode	0.015	0.983(0.955,1.012)	0.277
	Oxalobacter	11	Inverse variance weighted	0.005	1.011(1.001,1.021)	0.036
			MR Egger	0.024	1.019(0.971,1.069)	0.461
			Simple mode	0.009	1.011(0.994,1.029)	0.242
			Weighted median	0.006	1.012(1.000,1.024)	0.058
			Weighted mode	0.008	1.010(0.994,1.027)	0.250
	Paraprevotella	13	Inverse variance weighted	0.005	1.011(1.001,1.021)	0.031
			MR Egger	0.016	0.980(0.949,1.012)	0.237
			Simple mode	0.011	1.014(0.992,1.037)	0.228
			Weighted median	0.007	1.009(0.996,1.022)	0.185
			Weighted mode	0.011	1.010(0.989,1.031)	0.368
	Prevotella7	11	Inverse variance weighted	0.004	1.009(1.001,1.017)	0.022
			MR Egger	0.024	0.989(0.943,1.037)	0.665
			Simple mode	0.009	1.006(0.989,1.024)	0.505
			Weighted median	0.006	1.009(0.998,1.020)	0.129
			Weighted mode	0.009	1.007(0.989,1.024)	0.470
	Rikenellaceae RC9 gut group	11	Inverse variance weighted	0.004	1.010(1.002,1.018)	0.015
			MR Egger	0.025	0.997(0.949,1.046)	0.898
			Simple mode	0.008	1.011(0.996,1.027)	0.186
			Weighted median	0.005	1.010(1.000,1.020)	0.040
			Weighted mode	0.008	1.011(0.995,1.027)	0.217

**Table 5:** Mendelian randomization result of casual effects between gut microbiome and the risk of depression.

Group	Bacterial	Nsnp	Methods	SE	OR (95% CI)	P-value
Phylum	Lentisphaerae	9	Inverse variance weighted	0.002	1.004(1.000,1.007)	0.035
			MR Egger	0.007	1.008(0.995,1.021)	0.261
			Simple mode	0.004	1.001(0.994,1.009)	0.725
			Weighted median	0.002	1.003(0.998,1.007)	0.266
			Weighted mode	0.004	1.002(0.995,1.009)	0.626
Class	Lentisphaeria	12	Inverse variance weighted	0.002	1.004(1.001,1.008)	0.019
			MR Egger	0.007	1.009(0.996,1.022)	0.227
			Simple mode	0.004	1.002(0.995,1.010)	0.533
			Weighted median	0.002	1.003(0.998,1.007)	0.263
			Weighted mode	0.004	1.003(0.995,1.010)	0.527
Order	Actinomycetales	5	Inverse variance weighted	0.003	1.007(1.000,1.013)	0.047
			MR Egger	0.009	1.011(0.993,1.030)	0.320
			Simple mode	0.005	1.007(0.997,1.018)	0.255
			Weighted median	0.004	1.007(1.000,1.015)	0.047
			Weighted mode	0.005	1.007(0.997,1.017)	0.241
	Victivallales	8	Inverse variance weighted	0.007	1.004(1.001,1.008)	0.019
			MR Egger	0.002	1.009(0.996,1.022)	0.227
			Simple mode	0.007	1.002(0.995,1.010)	0.563
			Weighted median	0.004	1.003(0.998,1.007)	0.265
			Weighted mode	0.002	1.002(0.995,1.010)	0.541
Family	Actinomycetaceae	5	Inverse variance weighted	0.003	1.007(1.000,1.013)	0.046
			MR Egger	0.009	1.011(0.993,1.030)	0.319
			Simple mode	0.005	1.007(0.996,1.018)	0.262
			Weighted median	0.004	1.007(1.000,1.014)	0.046
			Weighted mode	0.005	1.007(0.997,1.017)	0.244
	Peptostreptococcaceae	14	Inverse variance weighted	0.002	1.005(1.001,1.009)	0.014
			MR Egger	0.005	1.009(0.992,1.010)	0.865
			Simple mode	0.005	1.009(0.100,1.019)	0.079
			Weighted median	0.003	1.006(0.100,1.012)	0.065
			Weighted mode	0.004	0.100(0.991,1.009)	0.981
	Streptococcaceae	14	Inverse variance weighted	0.003	0.993(0.988,0.999)	0.013
			MR Egger	0.011	0.984(0.963,1.007)	0.194
			Simple mode	0.007	0.992(0.979,1.005)	0.251
			Weighted median	0.004	0.993(0.986,1.001)	0.069
			Weighted mode	0.007	0.993(0.980,1.007)	0.330
Genus	Eubacterium ventriosum group	15	Inverse variance weighted	0.002	1.006(1.001,1.011)	0.017
			MR Egger	0.011	1.023(1.002,1.045)	0.052
			Simple mode	0.005	1.012(1.001,1.023)	0.049
			Weighted median	0.003	1.008(1.002,1.015)	0.010
			Weighted mode	0.005	1.011(1.000,1.022)	0.060
	Ruminococcus gnavus group	12	Inverse variance weighted	0.002	1.004(1.001,1.008)	0.025
			MR Egger	0.009	1.009(0.991,1.027)	0.332
			Simple mode	0.004	1.007(0.100,1.015)	0.084
			Weighted median	0.002	1.006(1.002,1.011)	0.008
			Weighted mode	0.004	1.007(1.000,1.015)	0.073
	Ruminiclostridium6	16	Inverse variance weighted	0.002	1.005(1.002,1.009)	0.006
			MR Egger	0.005	0.999(0.990,1.009)	0.860
			Simple mode	0.006	1.012(1.001,1.024)	0.050
			Weighted median	0.003	1.005(0.999,1.010)	0.084
			Weighted mode	0.005	0.999(0.988,1.010)	0.856
	Streptococcus	15	Inverse variance weighted	0.003	0.993(0.986,0.997)	0.003
			MR Egger	0.009	0.969(0.952,0.986)	0.002
			Simple mode	0.006	0.989(0.977,1.002)	0.122
			Weighted median	0.003	0.992(0.985,0.999)	0.020
			Weighted mode	0.006	0.990(0.978,1.001)	0.095
